# Grain-to-Grain Interaction Effect in Polycrystalline Plain Low-Carbon Steel within Elastic Deformation Region

**DOI:** 10.3390/ma14081865

**Published:** 2021-04-09

**Authors:** Hai Qiu, Rintaro Ueji, Yuuji Kimura, Tadanobu Inoue

**Affiliations:** Research Center for Structural Materials, National Institute for Materials Science, 1-2-1 Sengen, Tsukuba 305-0047, Ibaraki, Japan; Ueji.Rintaro@nims.go.jp (R.U.); Kimura.Yuuji@nims.go.jp (Y.K.); Inoue.Tadanobu@nims.go.jp (T.I.)

**Keywords:** Young’s modulus, elastic strain, polycrystalline steel, crystal orientation, grain, digital image correlation

## Abstract

A grain is surrounded by grains with different crystal orientations in polycrystalline plain low-carbon steel. The grain is constrained by its adjacent grains in the tension process. The interaction of the grain with the adjacent grains was investigated within the elastic deformation region. The following results have been obtained: (1) the Young’s modulus of a grain without consideration of grain-to-grain interaction is denoted as the inherent Young’s modulus; when the inherent Young’s modulus of a grain is equal to the Young’s modulus of the bulk material, there is almost no interaction between the grain and its adjacent grains; when a grain has a great difference between its inherent Young’s modulus and the Young’s modulus of the bulk material, its grain-to-grain interactions increase significantly; (2) the grain-to-grain interaction is mainly caused by the difference in the inherent Young’s modulus between the grain and its adjacent grains; the misorientation angle between the grain and its adjacent grains has almost no effect on the grain-to-grain interaction.

## 1. Introduction

In crystalline materials, the elastic strain is small, and the elastic behavior is linear [1]. The relationship between stress and strain in the elastic deformation is described by Hooke’s law. In the case of simple tension, Hooke’s law simplifies into a directly proportional function, i.e., the tensile stress is proportional to the tensile strain. The proportionality constant in this relationship is called Young’s modulus [1].

A grain is an important microstructural unit, and its Young’s modulus depends on its crystal orientation [2,3,4,5]. Grains in crystalline materials have different crystal orientations; correspondingly, they have different Young’s moduli. The deformation of a grain is constrained by its adjacent grains, resulting in grain-to-grain interaction. Abdolvand et al. [6] measured the stress within grains in Zr and Ti polycrystals (CPZr and CPTi) with three-dimensional synchrotron X-ray diffraction in the plastic deformation region. In a significant fraction of grains, the stress along the loading axis was found to decrease during tensile plastic flow just beyond the macroscopic yield point [6]. They believed that the observed stress drop was caused by the grain-to-grain interactions, and this interaction effect was strong in the plastic deformation region. Grain-to-grain interaction strongly influences the mechanical properties of a polycrystal. However, the grain-to-grain interaction effect in the elastic deformation region was studied less. The concern of the present study is on the grain-to-grain effect in the elastic deformation region.

**Figure 1:**
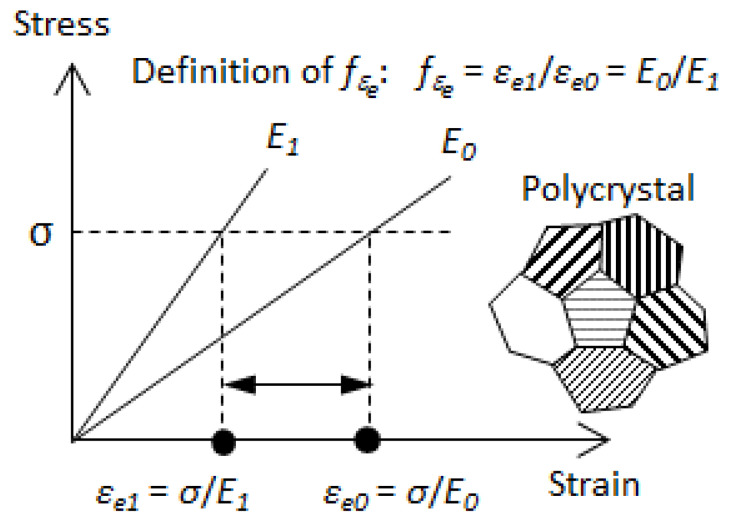
Schematic illustration of the effect of grain-to-grain interaction on a grain in terms of the difference in the elastic strain within the elastic deformation region. *E*_0_, Young’s modulus without grain-to-grain interaction; *E*_1_, Young’s modulus with grain-to-grain interaction; *ε_e_*, elastic strain of a grain.

As illustrated in Figure 1, the stress linearly varies with the strain within a grain in a polycrystal. The Young’s modulus of the grain is denoted as the inherent Young’s modulus (*E*_0_) when the grain deforms freely without constraint from its adjacent grains. When the grain is constrained by its adjacent grains during the tension process, its Young’s modulus will change from *E*_0_ to *E*_1_. At a given stress (*σ*), the elastic strain within the grain corresponding to *E*_1_ and *E*_0_ is *σ/E*_1_ and *σ/E*_0_, respectively. The two elastic strains (*ε_e_*_1_, *ε_e_*_0_) are different, and the difference between them is caused by the interactions between the grain and its adjacent grains. We define the elastic–strain ratio (*f_εe_*) as *f_εe_* = *ε_e_*_1_*/ε_e_*_0_ = *E*_0_*/E*_1_. *f_εe_* is independent of applied stress, and depends on the degree of grain-to-grain interaction. Therefore, it is rational to use it as a parameter to measure the effect of the interaction between a grain and its adjacent grains within the elastic deformation region: when *f_εe_* is equal to unity, it means that there is no grain-to-grain interaction; when *f_εe_* gradually deviates from one, the grain-to-grain interaction correspondingly increases significantly.

The *f_εe_* of a grain is determined by its Young’s modulus with and without grain-to-grain interactions (*E*_1_, *E*_0_). The inherent Young’s modulus of the grain (*E*_0_) is equal to the Young’s modulus of a single crystal with the same crystal orientation. The Young’s modulus of the single crystal can be measured via several methods which are suitable to the bulk material:

(1) *Hooke’s law* [1,7,8,9,10,11,12,13,14,15,16]: A simple tension (or simple compression) test is performed to obtain a stress–strain straight line within the elastic deformation region. The gradient of this line is the Young’s modulus.
(2) *Vibration test* [1,17]: Young’s modulus (*E*) is proportional to the square of the natural frequency (*f*) of the vibration of the bulk material ($E\propto f^{2}$). The *f* is obtained from a vibration test, and the *E* is derived from the relation between the *E* and *f*.
(3) *Sound velocity measurement test* [1]: The velocity of the longitudinal wave, $v_{l}$, depends on the Young’s modulus and the density of the bulk material, ρ, by the equation: $v_{l}=\sqrt{E/\rho }$. The Young’s modulus is determined by the above equation, in which $v_{l}$ is measured by the experiment.

The nanoindentation technique is a useful tool to measure the Young’s modulus of a local site. Its principle can be stated briefly as follows: a diamond indenter is pushed into the surface of the material, and the variation of load with the penetration depth of the indenter in the loading and unloading process is recorded; from the unloading curve, the Young’s modulus of the region where the indenter penetrated is obtained [2,3,4,5]. Many researchers have used the nanoindentation technique to measure the Young’s moduli of individual grains in polycrystals [2,3,4,5]. However, because the load–displacement curve in the nanoindentation test does not involve any information regarding grain-to-grain interaction, the obtained Young’s moduli were not the real ones of individual grains (*E*_1_) in the polycrystals, and they were close to the values of single crystals (*E*_0_) [5]. In addition to experimental measurements, *E*_0_ is available from the theoretical calculation based on the elastic constants [18].

As for the Young’s moduli of individual grains in a polycrystal, experimental measurements have not been reported in the literature due to the lack of an appropriate measurement method. In method (1) mentioned above (Hooke’s law), the stress–strain curve of a region of interest represents the real response of the region in a simple tension process, and, naturally, the effect of grain-to-grain interaction is involved. Therefore, this measurement method fits not only for the bulk material but also for local sites (e.g., individual grains) in a polycrystal. To obtain the stress–strain curve of a grain in a polycrystal, the key point is how to accurately measure the deformation behavior of individual grains.

Digital image correlation (DIC) is a non-contact measurement technique, established in the 1990s, for measuring the displacement and strain field on the surface of an object [19]. To perform DIC with high accuracy, a high-contrast random and dense speckle pattern is required [20]. For different scale lengths, different speckle patterns should be used [21]. DIC was used to measure the macrostrain of a bulk material, and the Young’s modulus of the bulk material was successfully determined from the obtained stress–strain curve [7,8,9,10,11,12,13,14,15,16]. Recently, research was carried out to determine appropriate experimental conditions, such as subset size and speckle, for micro- and mesoscale DIC [20,21,22,23,24]. It has been shown that, if the appropriate experimental setup and speckle are available, it is possible to measure the microstrain at the grain scale [24].

In this study, the concrete focuses are as follows: (1) we used micro-DIC to obtain the stress–strain curves of individual grains in a polycrystal within a macroscopic elastic deformation region, and from the experimental results, the corresponding Young’s moduli (*E*_1_) were determined; (2) the interaction between a grain and its adjacent grains within the elastic deformation region was evaluated in terms of the elastic–strain ratio (*f_εe_*), and the factors determining the value of *f_εe_* were revealed.

## 2. Materials and Methods

Individual grains in polycrystals were our main concern. To accomplish our purpose, an ideal polycrystal is expected, in which grains are equiaxial, their crystal orientations are random, and there are no additive effects (such as a second phase, precipitates, inclusions, etc.). In Ref. [24], a polycrystalline plain low-carbon steel with 0.05C-0.008Mn-0.002Si (in wt %) was produced. Its upper yield strength (*σ_up.ys_*) was 320 MPa, and its Young’s modulus was 207 GPa. It was mainly composed of ferrite (average grain size 30.4 µm) as well as a little pearlite (volume fraction 0.8%). The morphology and crystal orientations of ferrite grains were examined with the electron back-scattered diffraction (EBSD), and it was found that ferrite grains were equiaxial and that their crystal orientations were randomly distributed. This nearly ideal polycrystal with a body-centered cubic structure was used in this study.

**Figure 2:**
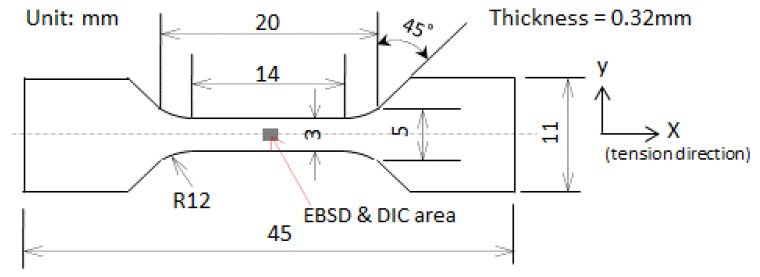
Specimen size.

**Figure 3:**
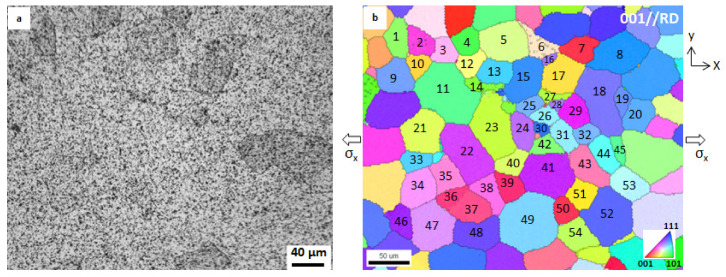
(**a**) Speckle on the etched front surface, (**b**) IPF (inverse pole figure) map corresponding to (**a**). The grains are numbered for digital image correlation (DIC). *σ_x_*, uniaxial remote stress.

A tension specimen (Figure 2) was machined. A simple tension test was carried out on the specimen at room temperature and at a crosshead speed of 0.1 mm/min. The experimental setup is described in Ref. [24]. The specimen was pre-processed before the tension test as follows: (1) the front surface was polished, followed by etching with 1.5% nital to expose the grain boundaries; (2) the crystal orientations around the center of the front surface were examined with EBSD; (3) a speckle pattern for DIC was prepared on the front surface. This pre-process has been described in detail elsewhere [24]. The region of interest was at the center of the front surface. The speckle and crystal orientations of grains around this region are shown in Figure 3a,b, respectively. The positions of Figure 3a,b match each other. The grains are numbered in Figure 3b, and they are the concern of the present study.

Two assumptions were proposed for obtaining the stress–strain curves of individual grains: (1) the stress imposed on each grain is composed of macroscopic stress, grain-to-grain interaction stress, and short-range dislocation stress [25]; it is assumed that grain-to-grain interaction stress and short-range dislocation stress are small in the elastic deformation region and that they can be neglected; the macroscopic stress imposed on each grain is uniform and is given by the load divided by the area of cross section; (2) the induced average strain within a grain is the average strain over the area enclosed by its grain boundary, as shown in Figure 3b. This average strain was obtained via 2D-DIC.

The deformation process of the region (shown in Figure 3) on the front surface was continuously recorded using a camera (2448 pixel × 2048 pixel, i.e., 0.41 mm × 0.34 mm). The DIC operation was performed on the digital images taken in the tension process by software of VIC-2D (subset size: 9 pixel × 9 pixel (4.8 µm × 4.8 µm); step: 5 pixel (1.7 µm)) to determine the displacement and strain filed within the grains. In the DIC operation, the displacement uncertainty is 0.02 pixel.

The obtained experimental Young’s modulus of a grain (*E_exp_*) involves the effect of grain-to-grain interaction. The Young’s modulus, *E_hkl_*, of a cubic grain with crystal orientation [*hkl*] without grain-to-grain interaction is given by [18].

(1) $$\frac{1}{E_{hkl}}=s_{11}+\frac{\left(2s_{12}-2s_{11}+s_{44}\right)\left(k^{2}l^{2}+l^{2}h^{2}+h^{2}k^{2}\right)}{{\left(h^{2}+k^{2}+l^{2}\right)}^{2}}$$

Individual elastic compliances, *s*, are related to the elastic constants, *c*, as:

(2) $$\{\begin{array}{c} s_{11}=\frac{c_{11}+c_{12}}{c_{11}^{2}+c_{11}c_{12}-2c_{12}^{2}}\\ s_{12}=\frac{-c_{12}}{c_{11}^{2}+c_{11}c_{12}-2c_{12}^{2}}\\ s_{44}=\frac{1}{c_{44}} \end{array}$$

The elastic constants for cubic crystal Fe are as follows: *c*_11_ = 231.4 GPa, *c*_12_ = 134.7 GPa, *c*_44_ = 116.4 GPa [18]. It is noted that the *E_exp_* and *E_hkl_* are in fact the *E*_1_ and *E*_0_ shown in Figure 1, respectively.

## 3. Results and Discussion

### 3.1. System Error of DIC Strain Measurement

The DIC technique has an inherent system error, which is dependent on the experimental setup, speckle, and DIC parameters [20,22]. In the measurement of a large strain, as compared with the applied strain, this system error is so small that it can be neglected. In the present study, the applied load in the simple tension test was limited below the upper yield strength, and the macroscopic longitudinal strain (*ε_x_*) was smaller than 1500 με. At this small strain level, attention must be paid to the accuracy of the DIC strain measurement.

The system error of the DIC strain measurement has been studied in terms of the strain noise and bias level using several digital images (image size: 0.41 mm × 0.34 mm) taken from the surface of an unloaded specimen [24]. The DIC operation was performed on these images to calculate the strain field. As no load was impacted, the real macroscopic strain should be zero. However, the calculated strain has an error due to the inherent system error. The standard deviation (STD) of the DIC strain field was defined as the strain noise level. The average strain of the whole strain field was taken as the calculated macroscopic strain, and the difference between it and the real macroscopic strain was defined as the bias level. For the DIC measurement conditions used in this study, the noise level is 80 με, and the bias level is 8 με [24]. It can be seen that the bias level is so small that the average strain obtained by the DIC is reliable at a microscale.

### 3.2. Young’s Modulus and Elastic-Strain Ratio of Individual Grains

**Figure 4:**
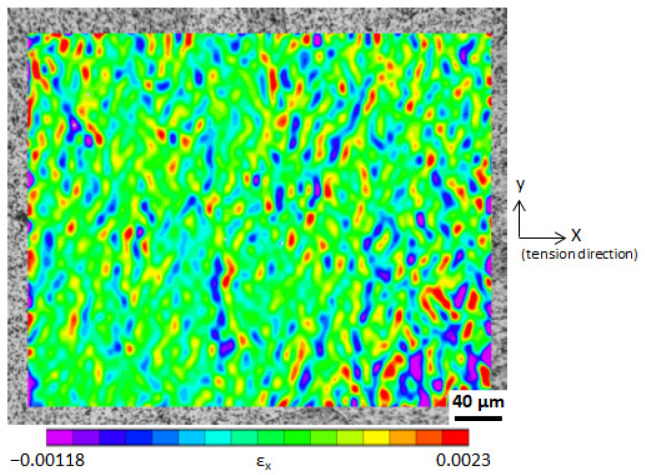
Two-dimensional longitudinal strain (*ε_x_*) field at an applied stress of 147 MPa.

The deformation process of the area shown in Figure 3 was recorded. A region of interest (ROI) was selected from the obtained digital images, and 2D-DIC was performed on the ROI. Figure 4 shows the longitudinal strain (*ε_x_*) field at an applied stress of 147 MPa whose ROI is about 390 μm × 320 μm. It was generally believed that the macroscopic strain within the elastic region is uniform at a macroscopic scale. However, Figure 4 shows that strain distribution is heterogeneous at a microscale. The applied stress (remote stress) is uniaxial. On a macroscopic scale, although the macroscopic stress in the parallel part of the specimen is uniform, in some local sites, e.g., cross-point of several adjacent grains, local stress is in a three-dimensional state and stress concentration occurs. As shown in Figure 4, even compression stress (compression strain) is present.

**Figure 5:**
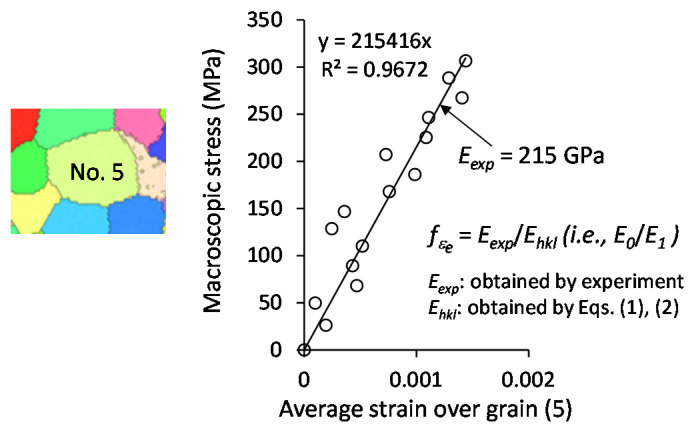
Determination of the Young’s modulus and elastic–strain ratio of grain (5) shown in Figure 3b.

Figure 4 covers more than 60 grains, in which the 54 gains selected (cf. Figure 3b) are involved. The grain boundaries can be identified in terms of the etched microstructure and the inverse pole figure (IPF) map; thus, the areas of individual grains can be selected as the ROIs, and the average strain over each grain can be determined from the strain fields of the ROIs. We took grain (5) (cf. Figure 3b) as an example. Grain (5) is surrounded by six grains. The average strains of grain (5), i.e., over the area enclosed by the grain boundaries, at sixteen stress levels were measured via 2D-DIC. The obtained strains as well as corresponding stresses are plotted in Figure 5. Fitting these experimental points with a straight line whose intercept is zero determines the experimental Young’s modulus (*E_exp_*) of grain (5). The Young’s moduli of other grains in Table 1 were determined in a similar way. The *E_exp_* and correlation coefficient (*R*^2^) are summarized in Table 1. There are three grains (No. 28, No. 30, No. 38) whose stress–strain curves did not show clear linearity. The subset is the unit of the DIC operation. The strain in the subset is assumed to be uniform; thus, the subset size represents the resolution of the DIC operation. The subset size used in the present study is 4.8 µm × 4.8 µm. The grain sizes of No. 28 and No. 30 are too small with respect to the subset size, resulting in a great error in the strain measurement. This may be the main reason for the nonlinearity of No. 28 and No. 30. However, the reason for No. 38 is unclear.

**Figure 6:**
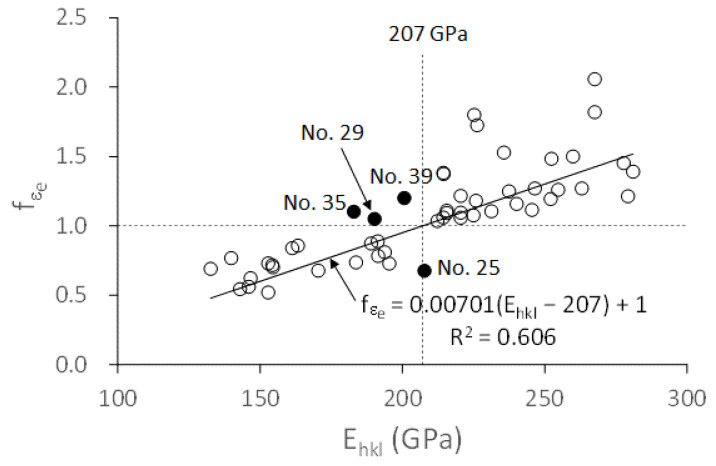
Elastic-strain ratio (*f_εe_*) of individual grains in Table 1. Vertical line, the Young’s modulus of the bulk material.

The *E_hkl_* for each grain was given by Equations (1) and (2), and the *f_εe_* was determined by the *E_hkl_* divided by the *E_exp_*. The *f_εe_* values of individual grains are shown in Figure 6. The Young’s modulus of the bulk material (vertical dotted line) is plotted in Figure 6. The experimental points are divided into two groups (the right and left groups) with respect to the vertical line. Except for the four abnormal points (solid circles) corresponding to grains of No. 25, No. 29, No. 35, and No. 39, the points in the right group have the values of *f_εe_* greater than one, while those in the left group have the values smaller than one. Although the scattering of data is substantial, a trend can be seen that when the *E_hkl_* moves away from the Young’s modulus of the bulk material in the right and left directions, the value of *f_εe_* deviates from one. The *f_εe_* has a linear relation with the *E_hkl_* as *f_εe_* = 0.00701(*E_hkl_* − 207) + 1, whose correlation coefficient (*R*^2^) is 0.606. This result indicates that when a grain’s inherent Young’s modulus is equal to the Young’s modulus of the bulk material, there is no grain-to-grain interaction effect on the grain; when the difference between a grain’s inherent Young’s modulus and the Young’s modulus of the bulk material increases, the grain-to-grain interaction effect increases significantly.

### 3.3. Factors Affecting the Elastic-Strain Ratio

**Figure 7:**
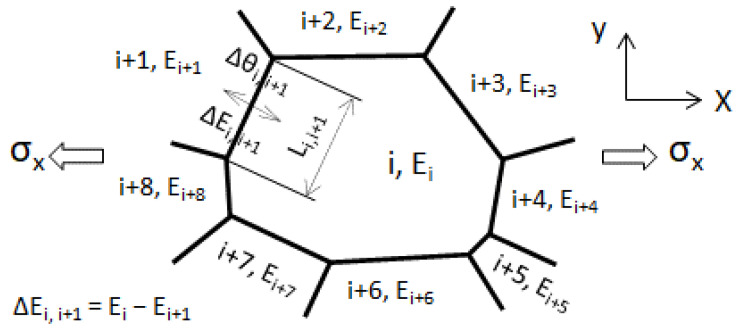
A diagram of a grain (numbered *i*) with its adjacent grains (numbered *i* + 1 to *i* + 8). E, inherent Young’s modulus; Δ*θ*, misorientation angle between two adjacent grains; Δ*E*, difference in the inherent Young’s moduli between two adjacent grains; L, grain boundary length. Suffix, grain number.

Figure 7 illustrates a grain with its adjacent grains subjected to stress along the *x*-axis (*σ_x_*). In a real material, a grain is constrained from three-dimensional directions (*x*-, *y*-, and *z*-axis). For simplification, grain *i* is assumed to be constrained within a two-dimensional plane (x-y plane) in Figure 7. Grain *i* is surrounded by several grains: eight grains (grain (*i* + 1) to grain (*i* + 8)) are plotted to illustrate. Grain *i* forms a couple with each adjacent grain, and each couple has the following characteristics:

(1) *Misorientation angle:* In a polycrystal, each grain has its specific crystal orientation; thus, the misorientation angle is present between two adjacent grains. The misorientation angle of grain *i* with grain (*i* + 1) to grain (*i* + 8) is denoted as Δ*θ_i,i_*_+1__,_ Δ*θ_i,i_*_+2_, …, Δ*θ_i,i_*_+8_. The misorientation angle of each grain in Table 1 with its adjacent grains can be determined from the IPF map shown in Figure 3b.
(2) *Difference in the inherent Young’s modulus:* The inherent Young’s modulus of grain *i* and its adjacent grains (grain (*i* + 1) to grain (*i* + 8)) is denoted as *E_i_*, *E_i_*_+1_, …, *E_i_*_+8_. We take grain *i* and grain (*i* + 1) as an example. The difference in the inherent Young’ moduli of grain *i* and grain (*i* + 1) is defined as Δ*E_i,i_*_+1_ = *E_i_* − *E_i_*_+1_. The grain-boundary length between the two grains is *L_i,i_*_+1_. The difference in the inherent Young’s moduli of grain *i* and other adjacent grains is defined in the same way.

**Figure 8:**
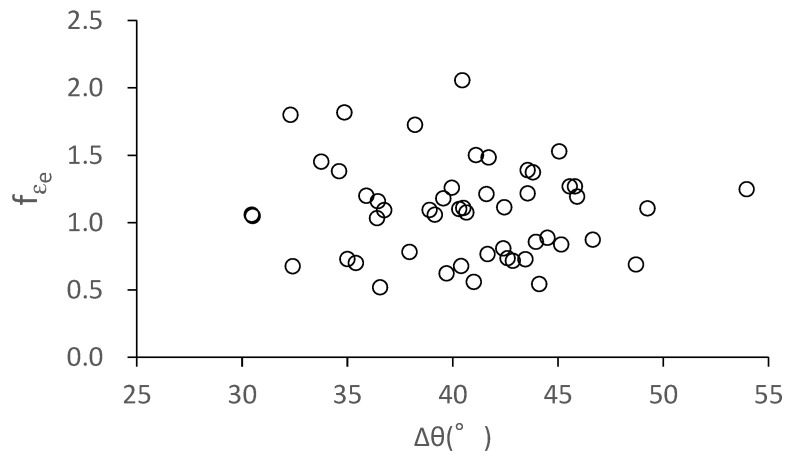
Correlation of average misorientation angle with the elastic-strain ratio. Δ*θ*, average misorientation angle of a grain with its adjacent grains; *f_εe_*, elastic–strain ratio.

The misorientation angle of grain *i* with its adjacent grains is one factor constraining grain *i*. We took the arithmetic average value of the misorientation angles ((Δ*θ_i,i_*_+1_ + Δ*θ_i,i_*_+2_ + … + Δ*θ_i,i_*_+8_)/8) as a statistical parameter, and investigated its effect on the *f_εe_*. The arithmetic average misorientation angle (Δ*θ*) of each grain in Table 1 with its adjacent grains and its *f_εe_* are plotted in Figure 8. No correlation between Δ*θ* and *f_εe_* is shown. This result indicates that the effect of the misorientation angle on the *f_εe_* can be neglected. It is noted that the misorientation angle of a grain with an adjacent grain in Figure 8 was measured at the center of the grain boundary between the two grains.

As shown in Figure 7, grain *i* is constrained by every adjacent grain. The extent of this constraint can be reflected in the absolute value of Δ*E_i,j_* (*j* = *i* + 1, *i* + 2, …, *i* + 8): a large value means a strong constraint, while a small value corresponds to a weak constraint. Some values of Δ*E_i,j_* are probably positive, and some are probably negative. Moreover, the grain boundary length of grain *i* with each adjacent grain is generally different. Therefore, the constraint from adjacent grains is extremely complicated, and it is difficult to evaluate the contribution of individual grains to the constraint. In the present study, we use the average value of Δ*E_i,i_*_+1_, Δ*E_i,i_*_+2_, …, and Δ*E_i,i_*_+8_ to describe the constraint from adjacent grains.

**Figure 9:**
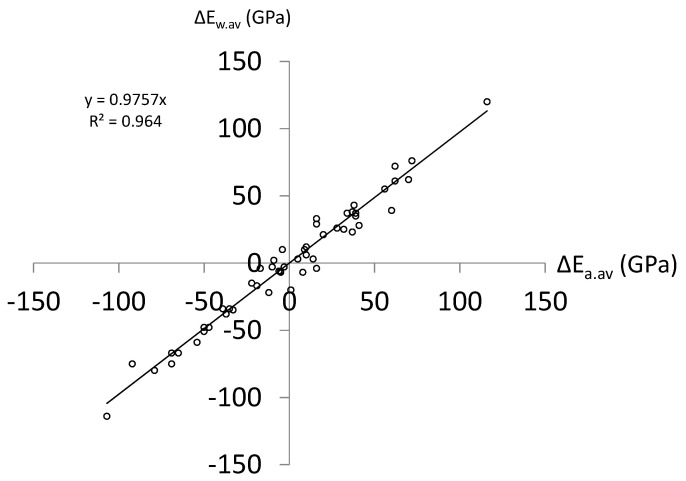
Arithmetic average value (Δ*E_a.av_*) and weighted average value (Δ*E_w.av_*) of the difference in the inherent Young’s modulus between a grain and its adjacent grains.

The arithmetic average value (Δ*E_a.av_*) of Δ*E_i,i+_*_1_, Δ*E_i,i+_*_2_, …, and Δ*E_i,i+_*_8_ is given by Δ*E_a.av_* = (Δ*E_i,i+_*_1_ + Δ*E_i,i+_*_2_ + … + Δ*E_i,i+_*_8_)/8. The weighted average value (Δ*E_w.av_*) of Δ*E_i,i+_*_1_, Δ*E_i,i+_*_2_, …, and Δ*E_i,i+_*_8_ is given by Δ*E_w.av_* = (*L_i,i+_*_1_Δ*E_i,i_*_+1_ + *L_i,i_*_+2_Δ*E_i,i_*_+2_ + … + *L_i,i_*_+8_Δ*E_i,i_*_+8_)/(*L_i,i_*_+1_ + *L_i,i_*_+2_ + … + *L_i,i_*_+8_). Δ*E_w.av_* seems to be more rational than Δ*E_a.av_* because the effect of the grain-boundary length of individual grains is involved. The Δ*E_a.av_* and Δ*E_w.av_* of all of the grains in Table 1 are calculated, and they are plotted in Figure 9. The Δ*E_w.av_* is nearly proportional to the Δ*E_a.av_* for the steel used. This means that either Δ*E_a.av_* or Δ*E_w.av_* is rational. It is noted that this conclusion was obtained from a nearly ideal polycrystal in which grains were uniform and equiaxial. Based on this result, we used the arithmetic average value instead of the weighted average value in the present study due to its convenience.

As illustrated in Figure 7, grain *i* is tensioned along the *x*-axis, and it deforms mainly along the *x*-axis. Grains (*i* + 1), (*i* + 8), (*i* + 7), (*i* + 3), (*i* + 4), and (*i* + 5) accommodate the deformation of grain *i* along the *x*-axis; thus, they contribute greatly to the constraint on grain *i*. In contrast to the deformation along the *x*-axis, deformation along the *y*-axis is small. Grains (*i* + 2) and (*i* + 6) inhibit the deformation of grain *i* along the *y*-axis; accordingly, their contribution to the constraint is small. The constraint on grain *i* caused by the difference in the Young’s moduli between grain *i* and its adjacent grains can be described in two ways: ① constraint only from the *x*-axis, and ② constraint from both the *x*- and *y*-axis. The degree of constraint corresponding to ① and ② is evaluated in terms of Δ*E_x_*/*E_hkl_* and Δ*E_x,y_*/*E_hkl_*, respectively, and given by:

Δ*E_x_*/*E_hkl_* = (Δ*E_i,i_*_+1_ + Δ*E_i,i_*_+8_ + Δ*E_i,i_*_+7_ + Δ*E_i,i_*_+3_ + Δ*E_i,i_*_+4_ + Δ*E_i,i_*_+5_)/(6*E_hkl_*) (3)

Δ*E_x,y_/E_hkl_* = (Δ*E_i,i_*_+1_ + Δ*E_i,i_*_+8_ + Δ*E_i,i+_*_7_ + Δ*E_i,i_*_+3_ + Δ*E_i,i_*_+4_ + Δ*E_i,i_*_+5_ + Δ*E_i,i_*_+2_ + Δ*E_i,i_*_+6_)/(8*E_hkl_*) (4)

The negative (or positive) value of Δ*E_x_*/*E_hkl_* or Δ*E_x,y_*/*E_hkl_* indicates that adjacent grains have greater (or smaller) inherent Young’s moduli than grain *i*; in other words, grain *i* is surrounded by hard (or soft) grains.

**Figure 10:**
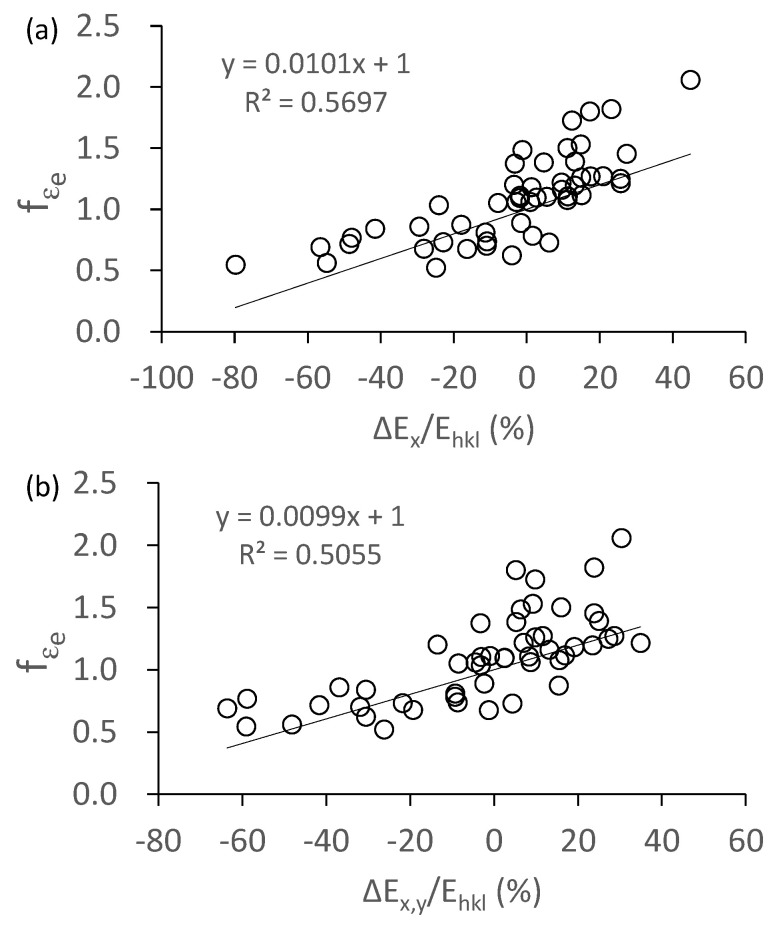
Effect of constraint degree on the elastic-strain ratio. (**a**) constraint along the *x*-axis, (**b**) constraint along both the *x*- and *y*-axis.

The *f_εe_* against Δ*E_x_*/*E_hkl_* and Δ*E_x,y_*/*E_hkl_* is plotted in Figure 10a,b, respectively. It can be seen from the values of the correlation coefficient (*R*^2^) that there is no great difference between Figure 10a,b. This indicates that either Δ*E_x_*/*E_hkl_* or Δ*E_x,y_*/*E_hkl_* is rational to describe the degree of the constraint. Figure 10 shows that when Δ*E_x_*/*E_hkl_* or Δ*E_x,y_*/*E_hkl_* is equal to zero, the *f_εe_* is equal to one; as the absolute value of Δ*E_x_*/*E_hkl_* (or Δ*E_x,y_*/*E_hkl_*) increases, the *f_εe_* gradually deviates from one. It means that for a grain, when there is no difference in the inherent Young’s moduli between it and its adjacent grains, no grain-to-grain interaction effect imposes on it; when this difference increases, grain-to-grain interaction increases significantly.

Although a linear trend is shown in Figure 10, its scatter is great. This is probably caused by two factors: (1) a grain is constrained from three-dimensional directions, but only two-dimensional constraint was considered in the present study; (2) a grain is surrounded by several grains, but each grain probably has a different effect, for example, some grains increase the degree of the grain-to-grain interaction, while others have the opposite effect. However, in the present study, we used the average value (Δ*E_x_*/*E_hkl_* or Δ*E_x,y_*/*E_hkl_*) to estimate the effect of the surrounding grains. This approach cannot accurately depict the effect of each grain, probably inducing a great error.

## 4. Conclusions

The interaction of a grain with its adjacent grains within the elastic deformation region of a polycrystalline plain low-carbon steel was investigated. The following conclusions were obtained:

(1) The DIC technique is a reliable tool to measure the Young’s modulus of individual grains in a polycrystal.
(2) The elastic–strain ratio—defined as the ratio of the elastic strain within a grain with grain-to-grain interaction to that without grain-to-grain interaction at a given stress—is a parameter independent of the applied stress. It can be used to evaluate the degree of grain-to-grain interaction within the elastic region.
(3) There is almost no constraint on the deformation of a grain whose inherent Young’s modulus is the same as the Young’s modulus of the bulk material. The grain-to-grain interaction effect is significant for a grain which has a great difference between its inherent Young’s modulus and the Young’s modulus of the bulk material.
(4) The interaction of a grain with its adjacent grains is mainly caused by the difference in the inherent Young’s moduli between the grain and its adjacent grains. The greater the difference, the greater the interaction. The average of the misorientation angles between the grain and its adjacent grains have almost no effect on the grain-to-grain interactions.

## Figures and Tables

**Table 1:** Young’s modulus and elastic–strain ratio of individual grains.

Grain No.	[hkl]			*E_hkl_* (GPa)	*E_exp_* (GPa)	*R* ^2^	*f_εe_*	Grain No.	[hkl]			*E_hkl_* (GPa)	*E_exp_* (GPa)	*R* ^2^	*f_εe_*
1	−2	5	17	153	209	0.6442	0.73	28	−7	−6	22	168	-	−2.665	-
2	−2	−3	3	268	147	0.8259	1.82	29	−2	1	4	190	181	0.9398	1.05
3	−7	8	11	260	173	0.7432	1.5	30	3	−3	8	187	-	−2.109	-
4	5	6	6	279	230	0.5752	1.21	31	−19	−5	26	214	155	0.8856	1.38
5	2	8	25	154	215	0.8725	0.72	32	−19	−5	26	214	202	0.8589	1.06
6	2	−3	3	268	130	0.7014	2.06	33	−8	−5	25	161	192	0.862	0.84
7	−1	−3	14	143	262	0.4016	0.55	34	1	−3	8	163	190	0.3082	0.86
8	5	6	9	246	194	0.785	1.27	35	−6	−3	13	183	166	0.7021	1.1
9	−11	−2	25	170	251	0.938	0.68	36	6	−14	21	215	197	0.7733	1.09
10	1	−3	3	236	154	0.7015	1.53	37	6	−14	21	215	194	0.8963	1.11
11	15	2	17	220	181	0.2365	1.22	38	1	2	6	160	-	−0.72	-
12	2	−2	15	140	182	0.9673	0.77	39	0	2	3	201	167	0.6794	1.2
13	−8	1	13	195	268	0.5746	0.73	40	4	−7	8	252	211	0.7984	1.19
14	9	−14	22	226	191	0.6547	1.18	41	−7	−5	9	255	202	0.9573	1.26
15	−8	−2	9	226	131	0.7602	1.73	42	13	−12	15	278	191	0.9617	1.45
16	−3	−11	13	225	125	0.9326	1.8	43	−1	−22	36	194	239	0.7243	0.81
17	1	−4	16	146	260	0.4253	0.56	44	−7	−2	22	154	220	0.0831	0.7
18	6	15	20	225	209	0.9326	1.08	45	−3	−1	12	147	235	0.1615	0.62
19	−11	−4	16	214	156	0.7553	1.37	46	−9	5	10	252	170	0.9535	1.48
20	−1	−13	22	191	215	0.8605	0.89	47	−7	−8	8	281	202	0.9903	1.39
21	4	7	10	231	209	0.8757	1.11	48	−1	10	13	212	205	0.9478	1.04
22	−8	−10	25	184	249	0.9442	0.74	49	14	−19	27	245	220	0.8862	1.12
23	1	0	27	133	192	0.9373	0.69	50	0	1	1	220	208	0.939	1.06
24	9	−9	23	191	244	0.5049	0.78	51	5	9	14	220	201	0.5511	1.1
25	2	−18	25	208	307	0.8213	0.68	52	19	1	33	189	216	0.9519	0.87
26	2	−5	17	153	293	0.4542	0.52	53	−19	8	22	237	190	0.7922	1.25
27	−12	15	19	263	207	0.8277	1.27	54	−14	−15	25	240	207	0.7853	1.16

## Data Availability

Data sharing is not applicable to this article.
